# The expanded footprint of the Deepwater Horizon oil spill in the Gulf of Mexico deep-sea benthos

**DOI:** 10.1371/journal.pone.0235167

**Published:** 2020-06-30

**Authors:** Michael G. Reuscher, Jeffrey G. Baguley, Paul A. Montagna

**Affiliations:** 1 Harte Research Institute for Gulf of Mexico Studies, Texas A&M University-Corpus Christi, Corpus Christi, Texas, United States of America; 2 Department of Biology, University of Nevada-Reno, Reno, Nevada, United States of America; University of Siena, ITALY

## Abstract

The 2010 Deepwater Horizon blowout off the coast of Louisiana caused the largest marine oil spill on record. Samples were collected 2–3 months after the Macondo well was capped to assess damage to macrofauna and meiofauna communities. An earlier analysis of 58 stations demonstrated severe and moderate damage to an area of 148 km^2^. An additional 58 archived stations have been analyzed to enhance the resolution of that assessment and determine if impacts occurred further afield. Impacts included high levels of total petroleum hydrocarbons (TPH) and polycyclic aromatic hydrocarbons (PAH) in the sediment, low diversity, low evenness, and low taxonomic richness of the infauna communities. High nematode to copepod ratios corroborated the severe disturbance of meiofauna communities. Additionally, barium levels near the wellhead were very high because of drilling activities prior to the accident. A principal component analysis (PCA) was used to summarize oil spill impacts at stations near the Macondo well, and the benthic footprint of the DWH oil spill was estimated using Empirical Bayesian Kriging (EBK) interpolation. An area of approximately 263 km^2^ around the wellhead was affected, which is 78% higher than the original estimate. Particularly severe damages to benthic communities were found in an area of 58 km^2^, which is 142% higher than the original estimate. The addition of the new stations extended the area of the benthic footprint map to about twice as large as originally thought and improved the resolution of the spatial interpolation. In the future, increasing the spatial extent of sampling should be a top priority for designing assessment studies.

## Introduction

The three-months-long uncontrolled release of crude oil from the Macondo MC252 oil well off the coast of Louisiana in the wake of the Deepwater Horizon (DWH) disaster had extensive effects on shallow water and deep-sea ecosystems alike [1]. A substantial percentage of the estimated 3.19 million gallons [2] of oil remained in the deep-sea [3]. Some of the crude oil was floating in mid-water plumes [4], where it aggregated with marine snow and settled on the seafloor [5]. Three important offshore and deep-sea habitats were studied as part of the Natural Resource Damage Assessment (NRDA) and the Gulf of Mexico Research Initiative (GoMRI): the pelagic realm [6, 7], deep-sea corals [8, 9], and the soft sediment benthos [10, 11, 12]. Each one of these habitats suffered from a loss of biodiversity caused by the smothering effects of oil as well as lethal and sublethal effects from a multitude of chemical compounds contained in crude oil [13]. In addition, the use of industrial oil dispersants, such as Corexit A9500^®^, increased the bioavailability of the oil [14] and introduced synergistic toxic effects to some of the organisms [15, 16, 17].

In the soft bottom benthic realm, an area of 172 km^2^ suffered from a loss of biodiversity, a decrease in the number of taxa, and an increased nematode to copepod (N:C) ratio caused by high levels of total petroleum hydrocarbons (TPH) and polycyclic aromatic hydrocarbons (PAH) [10, 18]. The most severe effects were found in the 24 km^2^ zone immediately surrounding the wellhead, where peak TPH and PAH levels were compounded by high barium concentrations from drill cuttings [10]. One year after the spill the hydrocarbon contamination and damage to the benthic fauna persisted, but signs of a mild recovery were detected [12, 19]. Four years after the spill there were indicators that meiofauna had recovered some, as the nematode to copepod ratio had decreased to background levels [20], but taxonomic richness was still significantly lower in the affected areas indicating no recovery from the primary damaging effects. Macrofauna still suffered from significantly lower diversity and taxa richness in 2014 [20].

During the original 2010 sampling, a total of 227 stations were sampled at water depths ranging from 10 m to 2767 m [21]. However, because of time constraints, only 58 stations were analyzed fully and used in the original estimate of the area of the deep sea that was damaged by the spill [10]. Priority was given to stations collected to the southwest of the wellhead because that is where the deep-sea plumes were detected. Shallow stations and stations to the north and northeast were ignored. Taking into consideration the heterogenous spatial distribution of the Deepwater Horizon fallout plume [22], the original footprint estimate of 148 km^2^ based on benthos [10] was likely an underestimation [23] because hopane in sediment yields estimates of 1,300 km^2^ [6] and 1,800 km^2^ [24], and radiocarbon in sediment yields estimates as large as 24,000 km^2^ [25]. For the present study, the footprint of the DWH oil on benthic infauna was reevaluated by including an additional 58 stations for a more comprehensive list of 116 stations sampled in 2010. With the newly included stations, this updated DWH footprint analysis extends over a larger area of the Gulf of Mexico and has improved spatial resolution.

## Materials and methods

Two research cruises (R/V Gyre, September 16—October 19, 2010, and R/V Ocean Veritas, September 24—October 30, 2010) commenced with the collection of deep-sea sediment samples, approximately two months after the Macondo oil well in Mississippi Canyon Block 252 (MC252) had been capped on 15 July 2010. Individual multicorer sample cores were set aside to study macrofauna, meiofauna, and sediment chemistry to assess damage that the hydrocarbon release had on the benthos. Marine benthic invertebrates were collected in the Gulf of Mexico, as part of NOAA's Office of Response and Restoration (ORR) contract DG133C06NC1729. The study area was the northern central and northeastern Gulf of Mexico, with the 116 sampling stations located on the continental shelf, the continental slope, and the Sigsbee Abyssal Plain. Sampling locations covered a depth range from 32 to 2767 m and a distance from the wellhead from approximately 0.3 km to 265 km (S1 Table). Further details about study design, sampling procedures, and sample processing were described previously [10, 19].

Macrofauna were identified to family level and meiofauna to higher taxonomic levels, ranging from order to phylum. Higher taxonomic levels have proved to be reliable surrogates for species level identification for the assessment of oil pollution impacts on benthic infauna communities [26]. Using higher taxonomic levels in environmental impact studies has the advantage that results may be obtained substantially faster. This was particularly important for the Deepwater Horizon disaster where evidence for the extent of the damage to the natural resources of the United States was crucial for the conclusion of legal proceedings and financial settlements [1, 27]. Measurements of macrofauna and meiofauna abundance (N) were standardized for an area of 1 m^2^ and 10 cm^2^, respectively. Taxonomic richness (R) of meiofauna was defined as number of taxa within the top 3 cm of sediment in a sampling core with 5.5 cm inner diameter. Taxonomic richness of macrofauna was defined as number of taxa in the top 10 cm of sediment, averaged across three cores with 10 cm inner diameter. Infauna diversity and evenness of distribution were calculated using Hill’s diversity number one (N1) [28] and Pielou’s evenness index (J´) [29]. For macrofauna N1 and J´ values of individual cores were averaged at each sampling station. All data are available in the S1 Table and the GRIIDC database [30].

Chemical contaminant and sediment grain size data were collected in the same multicorer drops as the infauna. Contaminant measurements were performed with the top 3 cm of sediment. Data were downloaded from the NRDA DIVER (Data Integration Visualization Exploration and Reporting) website https://www.diver.orr.noaa.gov/deepwater-horizon-nrda-data on 9 September 2016. This is the same data set reported on in the UAC (2010) report [31]. Methods for the chemical analyses are also described in the report and at http://www.nodc.noaa.gov/deepwaterhorizon/ship.html.

The publicly available bathymetry map “World Ocean Base” by Esri was obtained from http://www.arcgis.com/home/item.html?id=1e126e7520f9466c9ca28b8f28b5e500 on 4 December 2018. The seafloor contour shapefile was downloaded from the Gulf of Mexico Coastal Ocean Observation System (GCOOS) website at http://gcoos.org/products/topography/Shapefiles.html on 5 September 2018.

All biotic and chemical variables (X) were log-transformed using ln (X+1), except the N1 diversity index, which is already a log transformation of the Shannon diversity index H´. After transformation, all variables were standardized to a normal distribution with a mean of 0 and variance of 1 using the PROC STANDARD module contained in the SAS^®^ software suite. Raw and transformed data is provided in (S1 Table).

Principal components analysis (PCA) was used to classify the biological and environmental variables. PCA was performed using the PROC FACTOR module contained in the SAS software suite. The FACTOR analysis was run using the PCA method on the correlation matrix. The principal component scores, which represented the DWH oil spill, were used to categorize each station into five levels of impact, which were represented by different colors: red for severely impacted, orange for moderately impacted, yellow for possible minor impacts, and light and dark green for background levels. We used the Jenks natural breaks optimization (Goodness of Variance Fit) [32] classes from our 2013 analysis [10] and assigned the newly added stations to the different classes, based on their PC scores, which indicated oil spill impacts. The stations were plotted in ArcMap 10.6 in the respective colors of their impact level.

Empirical Bayesian Kriging (EBK) was used to predict the spatial extent of the DWH footprint. EBK is a powerful non-parametric interpolation method that does not assume normal distribution of the data [33]. This method has been successfully implemented for spatial analyses of the DWH impact in recent studies [e.g. 34]. The spatial prediction maps for the DWH impacts on the benthos were calculated in ArcMap 10.6, which has EBK implemented in its ArcGIS Geostatistical Analyst [35]. The same five classes that were described above for classification of sampling stations were implemented for classification of the spatially interpolated impacts. The areal extent of the impacts was determined in ArcMap with the areal measurement tool.

Non-metric multidimensional scaling (nMDS) was used to visualize similarities of macrofauna and meiofauna community structure among different sampling stations. Taxonomic abundance for both macrofauna and meiofauna were imported into Primer 7 and square-root transformed. This transformation is a standard procedure to increase the sensitivity of the analysis for rare taxa [36]. A Bray-Curtis similarity matrix was generated and used for the nMDS analysis. Individual stations were plotted as bubbles in the MDS ordinations. Bubble size visualizes PAH44 concentrations, colors represent the five impact levels, as described above. Stations with missing PAH44 concentrations were plotted as “X”. An analysis of similarity (ANOSIM) was performed to identify which stations have similar community structure. An analysis of similarity percentages (SIMPER) among species was performed to identify species contributing to community structure similarity among groups of stations.

## Results

**Fig 1:**
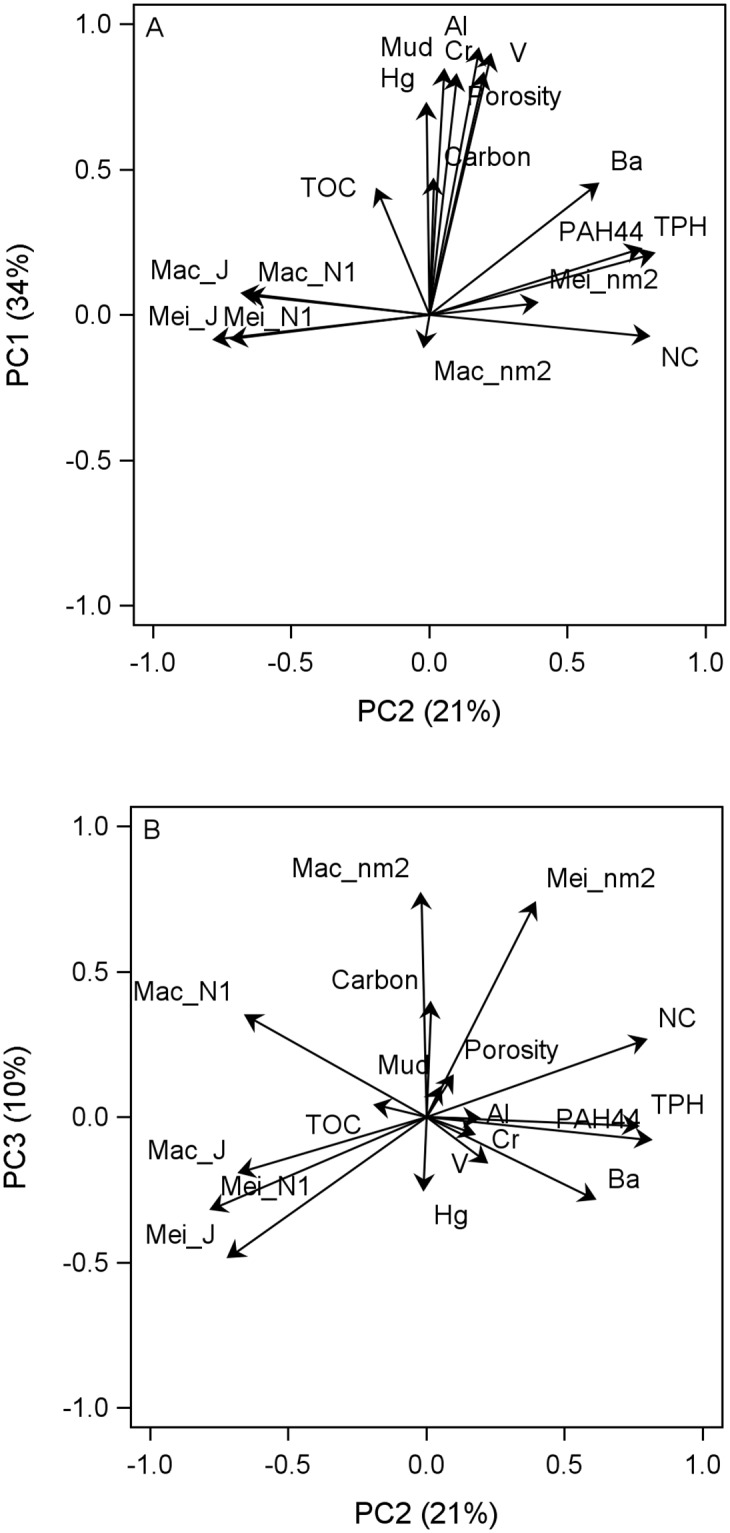
Variable loads for the Principal Components (PC) analysis for abiotic and biotic variables. A) PC1 and PC2 B) PC2 and PC3; variables included: aluminium (Al), barium (Ba), total carbon (Carbon), chromium (Cr), mercury (Hg), macrofauna evenness (Mac_J), macrofauna diversity (Mac_N1), macrofauna abundance (Mac_nm2), meiofauna evenness (Mei_J), meiofauna diversity (Mei_N1), meiofauna abundance (Mei_nm2), mud content (Mud), nematode to copepod ratio (NC), sum of 44 polycyclic aromatic hydrocarbons (PAH44), sediment porosity (Porosity), total organic carbon (TOC), total petroleum hydrocarbons (TPH), and Vanadium (Va).

**Fig 2:**
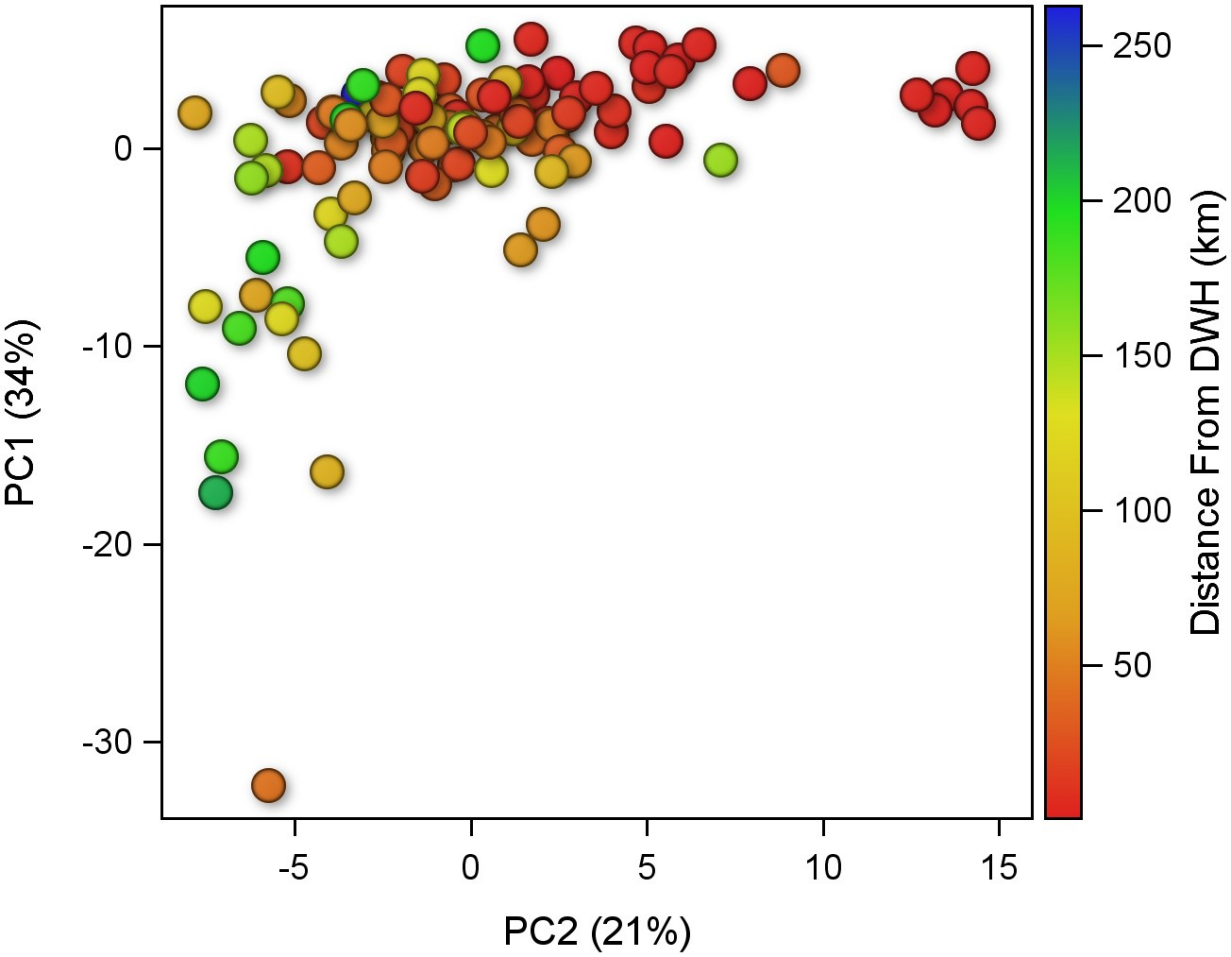
Station scores for PC1 and PC2 with symbols color-coded for distance from the wellhead. Red is nearest and blue is farthest.

The PCA analysis extracted eighteen orthogonal principal component (PC) factors. The first five PCs had Eigenvalues greater than 1 and accounted for 80% of the variance in the dataset. PC1 had an Eigenvalue of 6.1 and explained 34% of the variability. PC1 was driven by physical sediment qualities and the availability of carbon, as mud content, sediment porosity, and concentrations of carbon and total organic carbon (TOC) had highly positive loadings (Fig 1A). Additionally, PC1 was highly loaded by concentrations of several trace metals, including aluminum, chromium, mercury, vanadium, and, to a lesser degree, barium. None of the macrofauna or meiofauna metrics had high positive or negative loadings on PC1. PC2 had an Eigenvalue of 3.9 and explained 21% of the variability. PC2 represents DWH-related contaminations with high positive loadings by PAH44, TPH, and barium concentrations (Fig 1A). TPH and PAH44 are indicators of released petroleum, whereas barium is contained in drilling muds and fluids. Several faunal metrics were highly correlated with the DWH contaminants concentrations: meiofauna abundance and N:C ratio had high positive correlations, while diversity and evenness of both meiofauna and macrofauna had high negative correlations to the contaminants. In contrast, macrofauna abundance was unaffected, as its negative loading on PC2 was near zero. Distance from the wellhead was correlated with PC2 station scores (Fig 2). PC3 had an Eigenvalue of 1.9 and explained 10% of the variability. PC3 revealed correlations between the different faunal metrics and their relation to carbon concentrations. Carbon concentrations were positively correlated to abundance of meiofauna and macrofauna, as well as N:C ratios (Fig 1B). Conversely, evenness of community structure decreased with higher carbon concentration, in both meiofauna and macrofauna. Interestingly, diversity of macrofauna was positively correlated with carbon concentrations, while meiofauna diversity showed the opposite trend. PC4 had an Eigenvalue of 1.5 and accounted for 8% of the variability. PC5 had an Eigenvalue of 1.1 and explained 6% of the variability.

**Fig 3:**
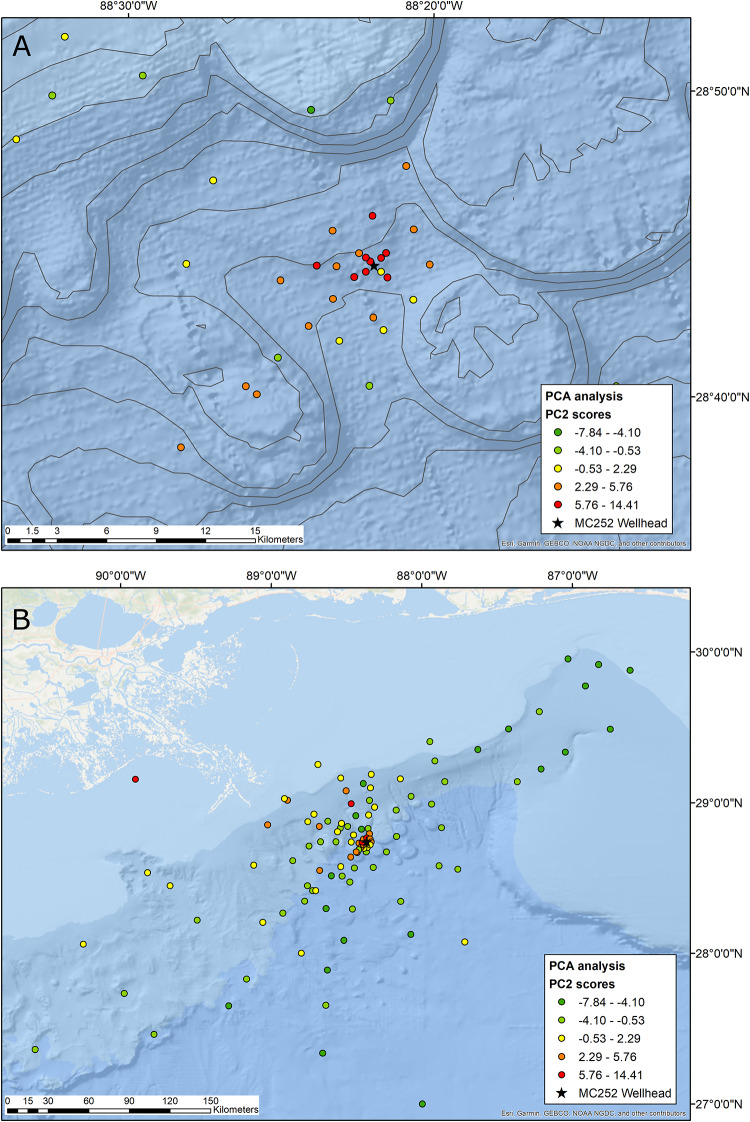
Sampling stations plotted by the color of their impact level based on PC2 scores. Red: high impact; orange: moderate impact; yellow: potential impact; light and dark green: background conditions. A) Stations near the MC252 wellhead. B) All sampling stations.

Seven of the nine stations located within the roughly 1 km radius (0.3–1.25 km) around the wellhead were classified as severely impacted in the original study [10] and retain that classification here (Fig 3a). These seven stations had high sediment concentrations of TPH (590–5,023 μg/g), PAH44 (143–1,181 ng/g), barium (789–12,700 μg/g), and very high N:C ratios (33–109). The high N:C ratio was caused by low copepod abundances at all the heavily impacted stations (n = 25.3–109.4 ind/10 cm^2^) and high numbers of nematodes at most impacted stations. The highest nematode abundance was detected at a heavily impacted station 1 km to the southeast of the wellhead (n = 8,504.8 ind/10 cm^2^). The impacted stations also had low taxonomic richness and diversity. The three most depauperate stations, located within a 0.65 km radius to the north of the spill, had only three meiofauna taxa present: nematodes, harpacticoids, and polychaetes. The highest value for meiofauna taxonomic richness at the seven highly impacted stations was six, compared to up to fourteen at unaffected sampling stations. Macrofauna showed similar trends with particularly low taxonomic richness (R = 4.3–14.0) and diversity values (N1 = 2.35–9.91) at highly impacted sampling stations, compared to maximum values at unaffected stations of R = 38.7 and N1 = 27.15, respectively. Two additional stations that were located 3 km from the wellhead were classified as severely impacted, even though their contaminant concentrations were lower and faunal metrics less impaired when compared to the seven stations described above (S1 Table). The closest moderately impacted station was located 1 km to the northwest of the wellhead. Six stations within a 3 km radius were also classified as moderately impacted. Moderate impacts from the DWH oil spill were detected as far as 15 km to the southwest and 6.25 km to the northeast. The moderately impacted stations typically had elevated contaminant concentrations and impaired faunal metrics, albeit less pronounced than the severly impacted stations.

Two heavily impacted stations had low TPH and PAH levels, indicating a different cause of disturbance. One of these stations was located almost 30 km north of the spill site, in a depth of 710 m (Fig 3B). Meiofauna and macrofauna at this sampling site were dominated by nematodes and cirratulid polychaetes, respectively. Thus, evenness and diversity values were among the lowest of all stations, while the N:C ratio was the second highest. A shallow station, 12 km off Grand Isle, Louisiana (Fig 3B) had the highest N:C ratio (381) and low values for macrofauna and meiofauna diversity and evenness. Twenty-seven stations were categorized as possibly impacted because they fell within the range of uncertainty (S1 Table). One of them was only 0.5 km to the southeast of the wellhead. TPH levels at this station were elevated (898 μg/g) but much lower compared to the other stations within a 1 km radius. Macrofauna abundance, richness, and diversity, as well as meiofauna abundance and richness were very low at this station, resembling the severely and moderately impacted stations near the wellhead. Conversely, values for N:C ratio, meiofauna diversity, and evenness of both meiofauna and macrofauna were comparable to stations with background conditions. It is noteworthy that TPH and PAH44 levels on the central shelf and slope are, with few exceptions, much higher than in the Eastern Gulf of Mexico. These higher concentrations may be caused by the abundant natural hydrocarbon seeps, oil exploration, or the deposition of DWH hydrocarbons. Sixty stations were representative of the natural background or pristine (light or dark green) category (S1 Table). Only one of these stations (LBNL4), located 7.5 km to the southwest of the spill site and nested about halfway between moderately impacted stations, had slightly elevated levels of TPH (183 μg/g). This station had a relatively diverse and evenly distributed faunal community with a very low N:C ratio of 1.4. All stations on the Florida and Alabama continental shelf and upper continental slope, which had not been included in our 2013 study, appeared to be unaffected by the oil spill. In general, the far eastern stations had very low TPH and PAH44 concentrations, presumably because of the scarcity of natural seeps and oil extraction activities.

**Fig 4:**
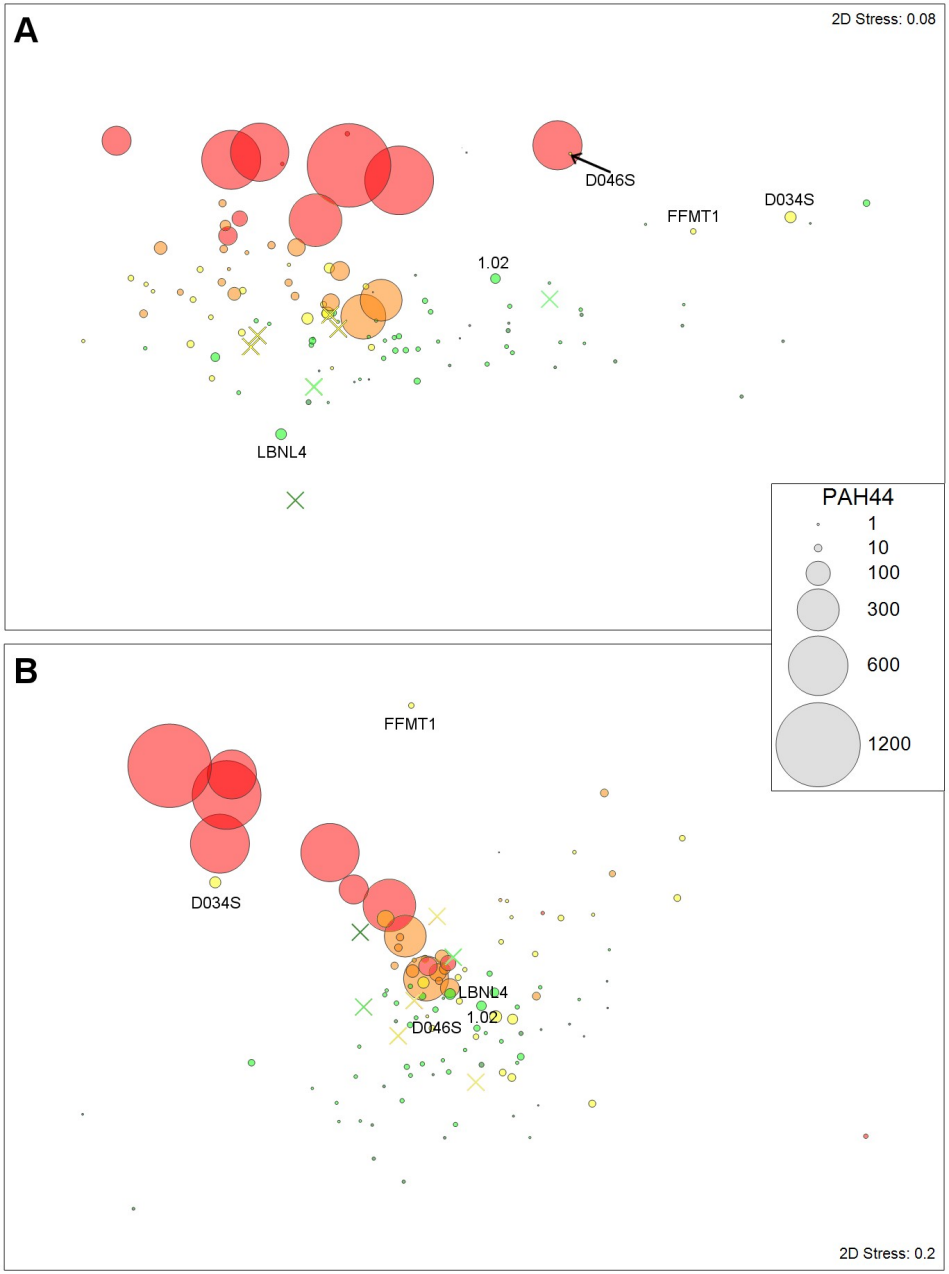
Multidimensional scaling (MDS) plots of benthic infauna community structure. Colors represent impact levels as defined in Figs 2 and 3, and the size of the bubbles represents PAH44 concentrations (ppb). An X indicates PAH44 concentration was missing. Station labels were included for those explicitly mentioned in the text. A) Meiofauna. B) Macrofauna.

The nMDS ordination plots indicate a correlation between PAH44 concentrations and the impact category zone of the individual sampling stations, as the largest bubbles were red and the smallest ones green (Fig 4). Both meiofauna and macrofauna nMDS plots had a gradual turnover in composition consistent with impact levels and PAH44 concentrations: the heavily impacted stations with high PAH levels were all located on top of the ordination plot, followed by orange and yellow stations, and the green stations at the bottom. There were differences in the community structure among the five categories for macrofauna (ANOSIM, p < 0.001) and meiofauna (ANOSIM, p < 0.001). Generally, the severely impacted group (1) was different, and the moderately and uncertain impact groups (2 and 3) were similar, and the pristine group (5) was always distinct (Table 1). The two heavily impacted stations, which did not have elevated TPH or PAH levels but very high N:C ratios, resembled the strongly impacted station in the meiofauna nMDS (Fig 4A). Nematode contribution to the meiofauna community declined from the severe to pristine station groups: severe = 83%, moderate = 69%, uncertain = 62%, no impact = 56%, and pristine = 53% (SIMPER). Another station (D046S), which was located 97 km southeast of the spill site and placed in the category “possibly impacted”, clustered with one of the heavily impacted stations (Fig 4A). Meiofauna communities at this station were dominated by nematodes (N:C ratio = 46), including very low numbers for taxonomic richness (5), diversity, and evenness. The lack of elevated TPH and PAH concentrations means that the nematode dominance at this station was unrelated to the DWH oil spill. Additionally, this station had a relatively rich, diverse, and taxonomically evenly distributed macrofauna community. In the macrofauna nMDS plot the uncertain-impact station (D034S), located only 0.5 km from the spill site, resembled the severely impacted stations (Fig 4B), because of its low taxonomic richness and diversity and its similarity in the number of dorvilleid and paraonid polychaetes (S2 Table). Station FFMT1, while not clustering with any severe or moderate impact station, was close to the top of the macrofauna nMDS plot (Fig 4B). This relatively shallow station in the Mississippi Trough had low values for abundance, richness, and diversity, but no evidence for DWH oil was found at this station. In general, dominance in macrofauna communities declined from severe to pristine station groups, as documented by the number of taxa that had a 70% cumulative contribution to the overall abundance: severe = 6 taxa, moderate = 12 taxa, uncertain = 14 taxa, no impact = 18 taxa, and pristine = 18 taxa (SIMPER, S2 Table).

**Table 1:** Results from ANOSIM test for differences in community structure among impact classification groups. Classification and color scheme as defined in Figs 3–5. Groups underlined are not different in community structure at the p = 0.05 level.

Taxa		Impact Classification Group			
A) Macrofauna					
Group	1	2	3	4	5
Color	Red	Orange	Yellow	Light Green	Green
Classification	Severe	Moderate	Uncertain	None	Pristine
B) Meiofauna					
Group	1	2	3	4	5
Color	Red	Orange	Yellow	Light Green	Green
Classification	Severe	Moderate	Uncertain	None	Pristine

**Fig 5:**
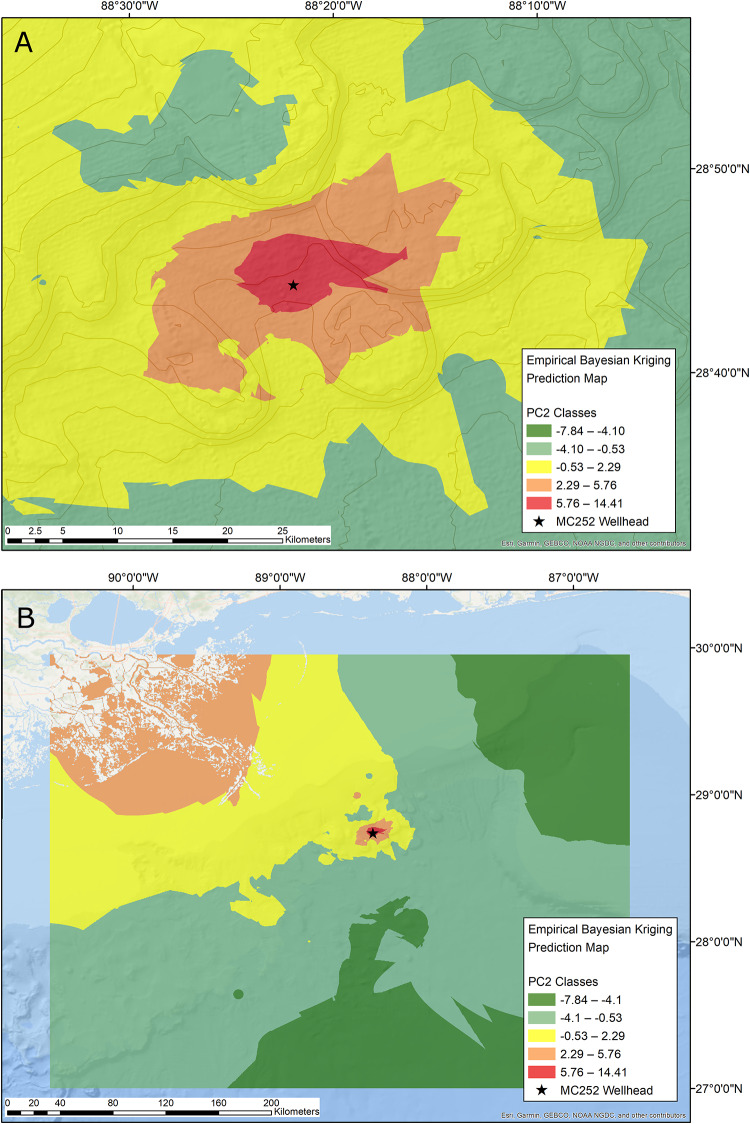
Benthic footprint maps determined by Kriging interpolation. Red: high impact; orange: moderate impact; yellow: potential impact; light and dark green: background conditions. A) Area near the MC252 wellhead. B) Entire sampling area.

The footprint of the Deepwater Horizon oil spill, as determined by Kriging interpolation analysis, was 321 km^2^ (Table 2). The most severe impacts were found in an area of approximately 58 km^2^ around the Macondo well (Fig 5A). The footprint area of 321 km^2^ includes only the red and orange zones around the Macondo oil well, but not the coastal areas further to the north, which were likely affected by hypoxia of the “dead zone” (Fig 5B). The shape of the impact zone around the wellhead was approximately elliptical, stretched in an approximate southwest to northeast direction.

**Table 2:** Dimensions of difference in impact zones between the 2013 and current studies. The colors refer to the impact zones in Figs 3–5. The predicted area of the orange zone excludes areas affected by causes other than the DWH oil spill, e.g., hypoxia or other drilling activity.

Zone (Map Color)	Based on Montagna et al. 2013		Based on current study	
	Area (km^2^)	Stations	Area (km^2^)	Stations
Severe Impact (Red)	24	8	58	11
Moderate Impact (Orange)	148	14	263	18
Uncertain Impact (Yellow)	13,497	13	23,063	27
No Impact (Light green)	33,760	16	58,785	40
Pristine (Dark green)	22,718	7	29,609	20
Total	70,147	58	111,778	116

## Discussion

The cluster of severely and moderately impacted stations around the Macondo oil well is a strong indication of the devastating effects of the DWH oil spill on the diversity of the benthic infauna. The locations of moderately impacted stations indicate that the DWH oil spread further to the northeastern and southwestern directions from the well than previously thought and causing damage to meiofauna and macrofauna assemblages in an area of approximately 321 km^2^. This estimate is considered conservative because it does not take into account the many patches of oil that are expected to be scattered throughout the northern Gulf of Mexico [37].

The loss of taxonomic richness at the affected stations was most likely caused by a combination of smothering [5] and the toxicity of contaminants, such as PAH44 and Barium [38]. This is evidenced by the PCA analysis, which indicates that DWH related contaminants (TPH, PAH44, and barium) were negatively correlated with taxonomic richness and diversity. Conversely, meiofauna abundance and N:C ratios were positively correlated with contaminant concentrations. The increase of opportunistic nematodes is a well-known phenomenon in disturbed and polluted habitats [39, 40, 41]. Additionally, many sensitive macrofauna taxa disappeared at affected stations [42], wheras tolerant taxa, such as dorvilleid polychaetes of the genus *Ophryotrocha*, thrived [12]. Our findings corroborate previous studies that found similar patterns of injuries in deep-sea corals [43] and infauna associated with these corals [11] near the DWH spill site.

The shallow station (1.20) off the coast of Grand Isle, Louisiana, USA, which had by far the highest N:C ratio among all stations, had no sign of oil contamination. The disturbance of the infauna was likely caused by hypoxic conditions, which are common along the Louisiana coast during summer months [44]. Hypoxia causes local extinction of sensitive organisms, while tolerant taxa may thrive [45]. The disturbance identified in the heavily impacted station 30 km north of the wellhead was also not related to DWH, as spill-related contaminants were not detected at high concentrations. Instead, there are records about three exploratory wells that were drilled between 0.7 km and 1.65 km from the station in 2005. Most likely meiofauna and macrofauna were disturbed during these operations and had not recovered five years later. It is known that drilling activities can cause long-term detectable effects in the benthos [41, 46].

The newly included stations in the northeastern Gulf of Mexico off Alabama and Florida did not exhibit signs of contamination. Their meiofauna and macrofauna communities were among the most species-rich and diverse. This leads to the conclusion that no widespread impacts on the benthic infauna of the Florida and Alabama shelf occurred after the DWH oil spill, even though other researchers found some evidence that the strong upwelling in 2010 did transport some of the oil to the Florida continental shelf [47].

The principal component, that was indicative of the oil spill changed from PC1 in our 2013 footprint assessment [10] to PC2 in our current study. This is an effect caused by the increase of stations included in the present study. The newly included stations increased the geographical coverage, the depth range, and many of the variables included in the PCA analysis. Therefore, the natural background accounted for more of the total variance than the DWH impacts. However, this does not mean that impacts of the DWH are considered less prevalent.

Compared to our 2013 study [10], our estimation of the area where the benthos was affected nearly doubled from 172 km^2^ to 321 km^2^ (Table 2). Estimations of area sizes of severe and moderate impacts increased from 24 km^2^ to 58 km^2^ and 148 km^2^ to 263 km^2^, respectively. This updated areal extent does not include the extensive moderately impacted coastal area, nor the small affected area 30 km north of the wellhead, which had other causes for their disturbances, as discussed above. The increased spatial resolution and the spatial interpolation method used in the present study make us confident that the expanded benthic footprint identified here is more realistic than our previous conservative estimation. However, the large size of the impacted area and the patchy distribution of the sequestered DWH hydrocarbons [22–24] makes it difficult to recreate the true spatial extent of the DWH impact on benthic infauna communities.

A total of 227 stations were sampled during the three 2010 cruises, but benthic macrofauna were sampled at only 171 of the stations during two of the cruises (R/V Gyre and R/V Ocean Veritas). Thus there are another 55 stations that remain unanalyzed. It is interesting to speculate if analyzing these additional samples might change the area of the footprint. We do not believe it would for two reasons. First, all but one of the the remaining stations are north of the spill area in shallow water (< 170 m) where neither the surface slick nor the deep sea plumes were present. The last station is very far to the northeast beyond the DeSoto Canyon. Second, the PAH44 concentrations at these stations was very low ranging from 7 to 177 ng/g (mean = 60, standard deviation = 42) [21].

We conclude that spatial analyses of environmental footprints should have a sampling design in place that covers a large area and includes many sampling stations to improve spatial resolution of the assessment. In the light of time and money constraints that are innate to any environmental assessment, we advocate that future complex environmental footprint assessments prioritize the optimization of spatial extent over multiple replicate samples from a single sampling station [48, 49]. Additionally, the substantial temporal variability of benthic communities in the northern Gulf of Mexico should be taken into consideration [50].

## Supporting information

S1 Table Station locations, data, and Principal Component (PC) scores. (XLSX)

S2 Table Macrofauna community similarity analysis (SIMPER results). (XLSX)
